# Supplementary material for: Risk Factor Analysis and a Predictive Model of Postoperative Depressive Symptoms in Elderly Patients Undergoing Video-Assisted Thoracoscopic Surgery
Source: Brain Sci. 2023 Apr 11;13(4):646. doi: 10.3390/brainsci13040646 (PMC10136581; doi:10.3390/brainsci13040646)
Supplement: Supplementary file 1 [file brainsci-13-00646-s001.zip › brainsci-2230279-supplementary.pdf]

brain sciences

Supplementary

Risk Factor Analysis and a Predictive Model of Postoperative Depressive Symptoms in Elderly Patients Undergoing Video-Assisted Thoracoscopic Surgery

Dinghao Xue [1,2,†], Xu Guo [2,†], Yanxiang Li [1,2,†], Zhuoqi Sheng [1], Long Wang [3], Luyu Liu [1,2], Jiangbei Cao [2], Yanhong Liu [2], Jingsheng Lou [2], Hao Li [2], Xinyu Hao [1,2], Zhikang Zhou [2] and Qiang Fu [2,*]

1 Medical School of Chinese PLA, Beijing, 100853, China.
2 Department of Anesthesiology, The First Medical Center, Chinese PLA General Hospital, Beijing, 100853, China.
3 Department of Pain Medicine, The First Medical Center, Chinese PLA General Hospital, Beijing, 100853, China.
* Correspondence: Dr_fuqiang@hotmail.com
† These authors contributed equally to this work.

Table S1. The Mini-Mental State Examination (MMSE).

<u>Instructions:</u> Ask the questions in the order listed. Score one point for each correct response within each question or activity.

Maximum Score	Patient's Score	Questions
5		"What is the year? Season? Date? Day of the week? Month?"
5		"Where are we now: State? County? Town/city? Hospital? Floor?"
3		The examiner names three unrelated objects clearly and slowly and then asks the patient to name all three of them. The patient's response is used for scoring. The examiner repeats them until the patient learns all of them, if possible. Number of trials:________
5		"I would like you to count backward from 100 by sevens."(93, 86, 79, 72, 65, ...) Stop after five answers."
3		"Earlier I told you the names of three things. Can you tell me what those were?"
2		Show the patient two simple objects, such as a wristwatch and a pencil, and ask the patient to name them.
1		"Repeat the phrase: 'No ifs, ands, or buts.'"
3		"Take the paper in your right hand, fold it in half, and put it on the floor." (The examiner gives the patient a piece of blank paper.)
1		"Please read this and do what it says." (Written instruction is "Close your eyes.")
1		"Make up and write a sentence about anything." (This sentence must contain a noun and a verb.)
1		"Please copy this picture."(The examiner gives the patient a blank piece of paper and asks him/her to draw the symbol below. All 10 angles must be present and two must intersect. 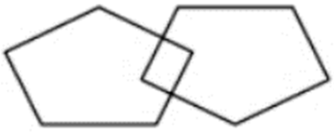
30		TOTAL

Table S2. The 9-item Patient Health Questionnaire (PHQ-9).
Over the past few days, how often have you been bothered by any of the following problems?

Items	Not at all	Several days	More than half of the days	Almost every day
1. Little interest or pleasure in doing things.				
2. Feeling down, depressed or hopeless.				
3. Trouble falling asleep, staying asleep, or sleeping too much.				
4. Feeling tired or having little energy.				
5. Poor appetite or overeating.				
6. Feeling bad about yourself—or that you're a failure or have let yourself or your family down.				
7. Trouble concentrating on things, such as reading the newspaper or watching television.				
8. Moving or speaking so slowly that other people could have noticed. Or, the opposite—being so fidgety or restless that you have been moving around a lot more than usual.				
9. Thoughts that you would be better off dead or hurting yourself in some way.				

Table S3. The 7-item Generalized Anxiety Disorder Questionnaire (GAD-7).

Over the past few days, how often have you been bothered by any of the following problems?

Items	Not at all	Several days	More than half of the days	Almost every day
1. Feeling nervous, anxious, or on edge.				
2. Not being able to stop or control worrying.				
3. Worrying too much about different things.				
4. Trouble relaxing.				
5. Being so restless that it's hard to sit still.				
6. Becoming easily annoyed or irritable.				
7. Feeling afraid as if something awful might happen.				
